# Simulation-based holistic education in physiotherapy interns to increase empathy toward older adults and individuals with disabilities

**DOI:** 10.1186/s12877-022-03500-x

**Published:** 2022-10-12

**Authors:** I.-Hsien Lin, Chien-Yung Wang, Yen-Nung Lin, Hung-Chou Chen, Li-Fong Lin

**Affiliations:** 1 Department of Physical Medicine and Rehabilitation, Wan Fang Hospital, Taipei Medical University, Taipei, Taiwan; 2 Graduate Institute of Injury Prevention and Control, Taipei Medical University, Taipei, Taiwan; 3 Taipei Neuroscience Institute, Taipei Medical University, Taipei, Taiwan; 4 Department of Rehabilitation, School of Medicine, College of Medicine, Taipei Medical University, Taipei, Taiwan; 5 Department of Physical Medicine and Rehabilitation, Shuang Ho Hospital, Taipei Medical University, New Taipei, Taiwan; 6 School of Gerontology and Long-Term Care, College of Nursing, Taipei Medical University, Taipei, Taiwan; 7 Neuroscience Research Center, Taipei Medical University, Taipei, Taiwan; 8 Research Center for Artificial Intelligence in Medicine, Taipei Medical University, Taipei, Taiwan

**Keywords:** Simulation, Holistic education, Intern, Empathy, Disability, Older adults

## Abstract

**Background:**

The proportion of older adults and individuals with disabilities in the general population increases each year. Thus, clinical clerkships designed for physiotherapy interns should provide more than simply knowledge and skills. For such interns to be able to handle the requirements of their future jobs, clerkships must enable them to develop empathy and positive attitudes toward patients. This study investigated the effect of simulation-based, holistic health-care education on physiotherapy interns’ empathy, attitudes, and knowledge.

**Methods:**

A parallel-group design. Thirty physiotherapy interns from a medical institution were enrolled as participants, with experimental and control groups each comprising 15 participants. Both groups received standard clinical training. However, the experimental group received an additional 3.5 h of simulation-based holistic health-care education. The Jefferson Scale of Empathy, Kogan’s Attitudes Toward Old People Scale, the Attitudes Towards the Elderly Scale, the Knowledge About Aging Scale, Knowledge of the Situation of Older People Scale, Perceptions of Working with Older People Scale, and Care Willingness Scale were used in a pretest and posttest.

**Results:**

After the intervention period, we observed substantial between-group differences of 6.4 points on the Jefferson Scale of Empathy (*p* = 0.001), 7.7 points on Kogan’s Attitudes Toward Old People Scale (*p* = 0.002), 3.5 points on the Attitudes Toward the Elderly Scale (*p* = 0.002), 2.5 points on Knowledge About Aging (*p* = 0.055), 4.5 points on the Knowledge of the Situation of Older People Scale (*p* < 0.001), and 2.1 points on Perceptions of Working with Older People Scale (*p* = 0.046).

**Conclusion:**

Simulation-based, holistic health-care education can significantly improve the empathy, knowledge, and attitudes of physiotherapy interns.

## Background

The World Health Organization (WHO) defines older individuals as those aged over 65 years. According to statistics released by the Ministry of the Interior, 3.804 million individuals were aged over 65 years in Taiwan at the end of April 2021 [1]; individuals aged over 65 years accounted for 16.4% of the total population, indicating Taiwan met the WHO’s definition of an aged society [1]. Older adults often develop degenerative diseases, such as degenerative arthritis, cataracts, and hearing loss, all of which affect their daily life functions and may even cause disability. The National Development Council (2020) estimated that Taiwan has approximately 760,000 individuals with disabilities. Moreover, the populations of older adults and individuals with disabilities are increasing each year. Older adults and individuals with disabilities often believe that they cannot receive dignified care because professional caregivers lack empathy and listening and communication skills [2]. Moreover, a clinical observation that physiotherapy students’ lack of empathy for older adults affects their care and treatment skills. By wearing simulation teaching aids and undergoing situational simulation training, students can experience the difficulties encountered by older adults and individuals with disabilities through experiential learning. Situational simulation increases perceptual awareness and induces emotions. Simulation training can enhance the cognitive and emotional benefits of instruction through internalized learning about different roles [3] and can improve the quality of care for older adults [4]. Therefore, whether scenario-based simulation training can enhance physiotherapy interns’ empathy warrants investigation [5].

Senior-year physiotherapy students in Taiwan are required to complete a hospital internship before graduating and taking the licensure exam they must pass before seeking a job. Physiotherapy education programs not only provide students with the clinical and technical skills they require to resolve diseases and promote health but also enhance their empathy toward older adults and individuals with disabilities. Physiotherapy internships emphasize holistic health care, which involves both patient-centered care after illness and the promotion of a holistic health-care system. Holistic health care includes physical, mental, social, and spiritual care. In addition to the provision of accurate diagnostic and treatment, practitioners of holistic health care should consider the convenience, safety, immediacy, appropriateness, comfort, and comprehensiveness of medical services. Through clinical simulation, students can estimate the effects of diseases from multiple perspectives (e.g., from the perspective of patients, their families, and society) and determine how they can provide comprehensive care by cultivating the knowledge, skills, attitudes, and effective and individualized care skills required in a professional workplace.

Studies have applied methods, such as a flipped classroom [6], guided self-study [7], gamification teaching [8], pain neuroscience education [9], digital learning [10, 11], Mask- ED™ simulation [12, 13], peer simulation programs [14], patient simulation techniques [15], and virtual reality [16], to facilitate the transfer and absorption of professional knowledge. However, no study has investigated the effects and benefits of holistic teaching on empathy. Physiotherapy interns are often required to care for older adults and patients with disabilities in the workplace. Traditionally, students learn relevant skills by observing and emulating how their clinical instructors conduct evaluations, implement interventions, and care for patients. However, the empathy developed by students who do so is still insufficient. Students can simulate the difficulties older adults encounter due to physical degeneration by participating in simulation courses designed from the perspective of older adults and individuals with disabilities, by wearing a simulation suit to perform daily tasks, and by observing and experiencing the physical inconveniences and the perceptions of older adults and individuals with disabilities. This critical thinking teaching strategy can enable students to perceive patients’ feelings and develop empathy; this in turn can assist them in applying their knowledge to provide better care on the basis of the internalized feelings they experienced during their observations and in providing appropriate treatment. Physiotherapy interns must integrate and utilize the knowledge they learn in school and in clinical practice. In addition, they should engage in care discussions conducted by a holistic and interdisciplinary team to enhance their knowledge, skills, and attitudes. A study established a simulation training model for medical and nursing students. Students who underwent aging simulation training exhibited improved attitudes, empathy, and willingness to provide care. However, the results of studies in which such training was employed remain unclear, potentially because of differences in intervention methods and times [17–20].

The effect of simulation-based teaching intervention on physiotherapy interns remains unclear. This study investigated whether simulation-based holistic care teaching intervention could enhance physiotherapy interns’ empathy, attitudes, knowledge, and willingness to care for older adults and individuals with disabilities in Taiwan.

## Methods

### Study design

This study was conducted as a single-blind, randomized, parallel study, and participants were randomly divided into two groups at a ratio of 1:1 by using the sealed envelope technique. A researcher who was not involved in the study presented the participants with sealed envelopes containing cards of different colors. Each participant was allocated to one of the two study groups on the basis of the color of the card inside the sealed envelope (white: control group; black: experimental group). Randomization was performed in accordance with CONSORT guidelines. Participants were selected on the basis of inclusion and exclusion criteria and whether they provided consent for participation in the study. The evaluator was blinded to the group allocation and the simulation-based holistic care teaching intervention that would be applied. This study was approved by the Institutional Review Board of Taipei Medical University (N202006001) and performed in accordance with the guidelines of the Declaration of Helsinki. All participants provided written informed consent before data collection began.

### Participants

**Fig. 1:**
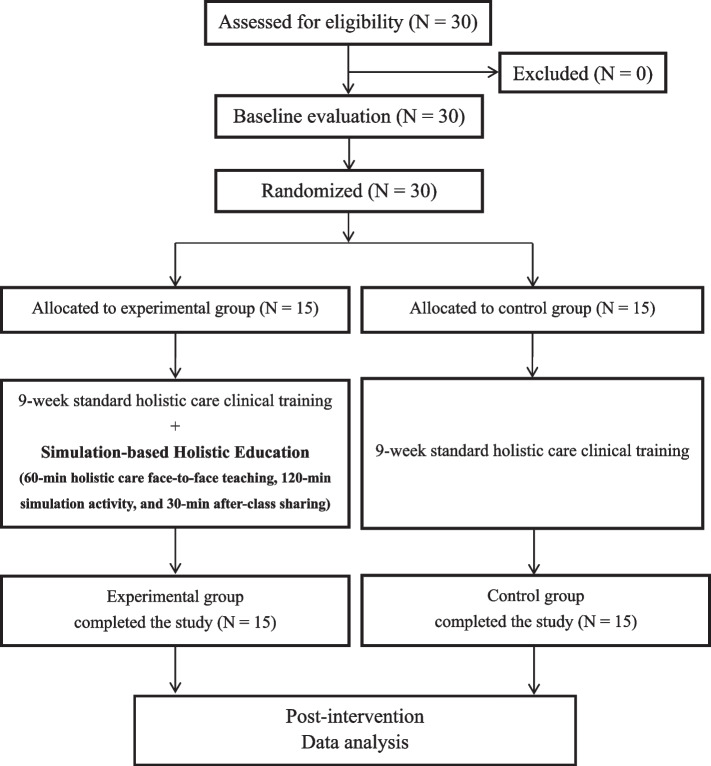
Study flow chart

The inclusion criterion was being older than 20 years. Students were excluded if they could not complete the questionnaire or had physical disabilities and injuries that prevented them from participating in simulation training. A pretest was conducted on the first day of participants’ internships. The participants were assigned to an experimental or control group. Both groups completed a standard clinical training program. However, from the ninth week onward, the experimental group received additional simulation-based holistic health-care education. In the tenth week, a posttest was conducted to compare the differences in empathy, knowledge, and attitudes toward older adults and individuals with disabilities between the groups. Figure 1 presents the processes of recruitment, intervention, assessment, and data analysis.

### Intervention

Simulation-based, holistic health-care education on older adults and individuals with disabilities was implemented in three stages. During the first stage, face-to-face, holistic health-care education session lasting 60-min were provided. During the second stage, a 120-min aged and disability simulation activity was conducted. Participants wore an age simulation suit (Yagami, Nagoya, Japan) and a hemiplegia simulation suit. The suits comprised a presbyopia eyeshade that created blurred vision, sound-blocking earmuffs that restricted hearing, a humpback simulation strap, restraint belts that limited elbow and knee movements, weight-bearing sandbags, restraint gloves that limited hand movements and tactile sensation, shoes with varying weights in each foot, a crutch, and a hemiplegic vest that limited upper-limb movements on one side. To simulate the daily life scenarios of older adults and individuals with disabilities, participants were asked to perform the following tasks at four checkpoints: (1) dining, (2) undressing and showering, (3) walking and stair climbing, and (4) shopping and registration (Table 1). Each participant played multiple roles through which they could observe and experience the physical and mental perspectives of older adults and individuals with disabilities. They played the roles of an activity participant, wearing a simulation suit to complete the designated tasks; an assistant, helping to maintain the safety of other activity participants; a supervisor, accompanying other activity participants who were completing the tasks; and an observer, viewing and attempting to empathize with the mental and physical inconveniences of older adults and individuals with disabilities. During the third stage, a 30-min, after-class, knowledge-sharing session was conducted, with participants sharing their reflections on the role-playing experience and their feelings regarding the roles they played during the simulation.

**Table 1:** Simulation activity tasks

Checkpoint	Task	Activity
**1**	**Dining**	Use your nondominant hand to pick up beans with chopsticks, scoop up round objects with a spoon, and drink water while wearing a simulation suit
**2**	**Undressing and showering**	Put on and take off clothes, move in and out of a bathtub, and shower while wearing a hemiplegia simulation suit
**3**	**Walking and stair climbing**	Stand up, push open a door, step over obstacles, and climb stairs while wearing a simulation suit
**4**	**Shopping and registration**	Register at a counter and remove a health insurance card and change out of a purse or wallet

## Outcome measures

The following information was collected on each participant: age, sex, religious beliefs, experiences of living with older adults, and participation in similar simulation training in the past. A pretest was conducted on the first day of participants’ internships. The experimental group received an additional 3.5 h of simulation-based, holistic health-care education during the ninth week, and a posttest was conducted in the tenth week (Fig. 1). Both the pretest and posttest were conducted by a physiotherapist who was blinded to the group allocation and, thus, the intervention received by each participant. The post-testing was independently completed in a paper-based manner by the participants.

### Primary outcome

The Jefferson Scale of Empathy–Health Profession Students (JSE-HPS) [21] was employed to measure participants’ empathy for older adults and individuals with disabilities. The scale comprised the following three dimensions: perspective taking (10 items), compassionate care (8 items), and standing in patients’ shoes (2 items). The 20 items were scored on a 7-point scale ranging from 1 (strongly disagree) to 7 (strongly agree), with total scores ranging from 20 to 140 points. A higher score indicated a higher level of empathy. The internal consistency was respectively determined to be 0.78 and 0.84 in two previous studies [22, 23].

### Secondary outcomes

The Chinese version of Kogan’s Attitudes Toward Old People Scale (KAOPS) was employed [24]. The scale comprised 34 items (17 reverse and 17 direct questions) [25] that were scored on a 6-point scale ranging from 1 (strongly disagree) to 7 (strongly agree); 4 points were assigned when a participant failed to respond to an item. Total scores ranged from 34 to 238 points. A higher score indicated a higher level of empathy. The Cronbach’s alpha for the scale was 0.82 [24]. The Attitudes Towards the Elderly Scale (ATES) was used to measure participants’ attitudes toward providing care for older adults. The scale comprised 23 items (11 reverse and 12 direct items). Direct items were scored on a scale ranging from 4 to 1, with 4, 3, 2, and 1 points indicating strongly agree, agree, disagree, and strongly disagree, respectively. Reverse items were scored on a scale ranging from 1 (strongly agree) to 4 (strongly disagree). Total scores ranged from 23 to 92 points. A higher score indicated a more positive attitude toward providing care. The Cronbach’s alpha was 0.84 for the scale [26].

The Knowledge About Aging Scale (KA) [26] was employed to estimate participants’ care knowledge. The scale comprised 50 items that were divided into four dimensions (i.e., physical and illness care, mental changes, social changes, and vital statistics). One point was assigned for a correct answer, and no point was assigned for a wrong or uncertain answer. Total scores ranged from 0 to 50 points. A higher score indicated a higher level of knowledge. The Cronbach’s alpha for the scale was 0.77. The Knowledge of the Situation of Older People Scale (KSOP) was adopted to estimate participants’ care knowledge. The scale comprised 13 items. One point was assigned for a correct answer, and no point was assigned for a wrong or uncertain answer. Total scores ranged from 0 to 13 points. A higher score indicated a higher level of knowledge. The Cronbach’s alpha coefficient for the KSOP Scale was 0.63 [27]. The Perceptions of Working with Older People Scale (PWOP) was used to measure participants’ perceptions of providing care to older adults. The 11 scale items were scored from 1 (strongly disagree) to 5 (strongly agree), with total scores ranging from 11 to 55 points. A higher score indicated more positive perceptions of providing care. The Cronbach’s alpha for the scale was 0.81 [28]. The Care Willingness Scale (CW) was used to measure participants’ willingness to provide care. The scale consisted of 13 items. The first 12 items comprised an equal number of reverse and direct questions. Direct items were scored from 4 to 1, with 4, 3, 2, and 1 points respectively indicating strongly agree, agree, disagree, and strongly disagree. Reverse items were scored on a scale ranging from 1 (strongly agree) to 4 (strongly disagree). Total scores ranged from 12 to 48 points. For item 13, participants were asked to describe how much they enjoyed taking care of older adults on a scale of 0 to 100, with a higher score indicating a higher willingness to provide care. The Cronbach’s alpha for the total scale was 0.78 [26].

### Statistical analysis

The recorded measurements were coded for data processing and analysis by using SPSS software (SPSS Statistics for Windows, Version 20.0, IBM, Armonk, NY). Continuous data are presented as means and standard deviations, and categorical data as numbers and percentages. The independent *t* test and chi-square test were performed to compare continuous and categorical data, respectively. Inferential statistics were determined using generalized estimating equations to determine the intervention effectiveness of the simulation course. The significance level was set to 0.05.

## Results

### Participants

A total of 30 participants met the inclusion criteria and provided consent to participate in this study. All participants completed the pretest and posttest. The experimental and control groups exhibited no significant differences in their demographic characteristics (*p* > 0.05; Table 2).

**Table 2:** Participant demographics

General characteristics	Experimental	Control	*p*
Participants (number)	15	15	
Age (Mean ± SD, years)	22.1 (1.4)	21.9 (2.0)	0.78
Sex (n, %)			
Men	9 (60%)	7 (46.7%)	0.45
Women	6 (40%)	8 (53.3%)	
Religious beliefs (n, %)			
Christianity	4 (26.7%)	0 (0%)	0.19
Taoism	8 (53.3%)	11 (73.3%)	
Buddhism	1 (6.7%)	2 (13.3%)	
Other	2 (13.3%)	2 (13.3%)	
Living with older adults (n, %)			
Yes	4 (26.7%)	3 (20%)	0.67
No	11 (73.3%)	12 (80%)	
Received similar courses in the past (n, %)	3/27		
Yes	1 (6.7%)	2 (13.3%)	0.54
No	14 (93.3%)	13 (86.7%)	

*SD* Standard Deviation

### Primary outcomes

#### Empathy

We compared the empathy levels between the experimental and control groups after the simulation-based, holistic health-care education intervention was implemented by using the JSE-HPS. The experimental group exhibited a 9.4-point improvement (from 42.6 ± 9.8 to 52.0 ± 11.9), whereas the control group demonstrated a 3.0-point improvement (from 42.1 ± 12.2 to 45.1 ± 12.9). Generalized estimating equations were used to analyze the interactions between the groups and time (pretest vs. posttest). Compared with the control group, the experimental group exhibited a significant 6.4-point (95% confidence interval [CI] = 2.5 to 10.2) improvement in the posttest relative to the pretest (*p* = 0.001). This finding suggests that the simulation-based, holistic health-care education intervention effectively enhanced empathy (Table 3).

**Table 3:** JSE-HPS, KAOPS, ATES, KA, KSOP, PWOP, and CW with interactions of group × time determined using GEE models

Variable (score)	Intervention	Experimental		Control		Between-group difference (Group^a^ × time^b^)				
		Mean (SD)	Within change	Mean (SD)	Within change	Estimate	SE	95% CI	wald	*p* value
JSE-HPS	Pre	42.6 (9.8)	9.4 (6.3)	42.1 (12.2)	3.0 (4.7)	6.4	1.9	2.5 to 10.2	10.3	0.001
	Post	52.0 (11.9)		45.1 (12.9)						
KAOPS	Pre	93.8 (10.7)	13.8 (7.1)	101.8 (13.7)	6.2 (7.1)	7.7	2.5	2.7 to 12.7	9.2	0.002
	Post	107.6 (7.4)		108.0 (13.1)						
ATES	Pre	61.7 (4.2)	5.4 (3.8)	63.0 (4.1)	1.8 (2.2)	3.5	1.1	1.2 to 5.6	9.7	0.002
	Post	67.1 (4.1)		64.8 (4.3)						
KA	Pre	37.3 (4.1)	5.0 (4.5)	35.7 (3.3)	2.3 (2.8)	2.5	1.3	0.1 to 5.1	3.7	0.055
	Post	42.3 (4.3)		38.2 (2.9)						
KSOP	Pre	3.1 (2.5)	5.2 (2.4)	3.7 (2.7)	0.7 (1.2)	4.5	0.7	3.1 to 5.8	43.7	< 0.001
	Post	8.3 (2.8)		4.4 (2.5)						
PWOP	Pre	36.4 (2.6)	3.6 (2.1)	35.4 (3.2)	1.3 (3.3)	2.1	1.0	0.1 to 4.1	3.9	0.046
	Post	40.0 (3.5)		36.7 (3.2)						
CW	Pre	31.9 (3.1)	3.4 (2.6)	29.6 (6.1)	3.8 (4.5)	0.5	1.3	2.1 to 3.0	0.1	0.723
	Post	35.3 (3.2)		33.4 (6.0)						

*SD* Standard Deviation, *JSE-HPS* Jefferson Scale of Empathy–Health Profession Students, *KAOPS* Kogan’s Attitudes Toward Old People Scale, *ATES* Attitudes Towards the Elderly Scale, *KA* Knowledge About Aging Scale, *KSOP* Knowledge of the Situation of Older People Scale, *PWOP* Perceptions of Working with Older People Scale, *CW* Care Willingness Scale, *SE* Standard Error, *CI* Confidence Interval

^a^Group: intervention vs. control group

^b^Time: pretest vs. posttest

### Secondary outcomes

#### Attitude

After the simulation-based, holistic health-care education intervention was implemented, we compared the attitudes toward older adults and individuals with disabilities of the experimental and control groups through their KAOPS scores. The experimental group exhibited a 13.8-point improvement (from 93.8 ± 10.7 to 107.6 ± 7.4), whereas the control group demonstrated a 6.2-point improvement (from 101.8 ± 13.7 to 108.0 ± 13.1). Regarding ATES scores, the experimental group exhibited a 5.4-point improvement (from 61.7 ± 4.2 to 67.1 ± 4.1), whereas the control group demonstrated a 1.8-point improvement (from 63.0 ± 4.1 to 64.8 ± 4.3). Generalized estimating equations were used to analyze the interactions between the groups and time (pretest vs. posttest). The KAOPS scores indicated that the experimental group demonstrated a significant 7.7-point (95% CI = 2.7 to 12.7) improvement in their posttest scores relative to their pretest scores (*p* = 0.002). Furthermore, the ATES scores indicated that the experimental group demonstrated a significant 3.5-point (95% CI = 1.2 to 5.6) improvement in their posttest scores relative to their pretest scores (*p* = 0.002). These findings indicate that simulation-based, holistic health-care education on older adults and individuals with disabilities effectively improves attitudes toward such individuals (Table 3).

#### Knowledge

After the simulation-based, holistic health-care education intervention was implemented, we compared the knowledge regarding older adults and individuals with disabilities of the two groups through their KA scores. The experimental group exhibited a 5.0-point improvement (from 37.3 ± 4.1 to 42.3 ± 4.3), whereas the control group demonstrated a 2.3-point improvement (from 35.7 ± 3.3 to 38.2 ± 2.9). The KSOP scores revealed that the experimental group exhibited a 5.2-point improvement (from 3.1 ± 2.5 to 8.3 ± 2.8), whereas the control group demonstrated a 0.7-point improvement (from 3.7 ± 2.7 to 4.4 ± 2.5). Generalized estimating equations were used to analyze the interactions between the groups and time (pretest vs. posttest). The KA scores revealed that the experimental group demonstrated a marginal 2.5-point (95% CI = 0.1 to 5.1) improvement in their posttest scores relative to their pretest scores (*p* = 0.055). The KSOP results indicated that the experimental group demonstrated a significant 4.5-point (95% CI = 3.1 to 5.8) improvement in their posttest scores relative to their pretest scores (*p* < 0.001). These findings indicate that the simulation-based, holistic health-care education on older adults and individuals with disabilities effectively enhanced knowledge regarding such individuals (Table 3).

#### Willingness to care

After the simulation-based, holistic health-care education intervention was implemented, we compared the willingness to provide care of the two groups through their PWOP scores. The experimental group exhibited a 3.6-point improvement (from 36.4 ± 2.6 to 40.0 ± 3.5), whereas the control group demonstrated a 1.3-point improvement (from 35.4 ± 3.2 to 36.7 ± 3.2). Regarding CW scores, the experimental group exhibited a 3.4-point improvement (from 31.9 ± 3.1 to 35.3 ± 3.2), whereas the control group demonstrated a 3.8-point improvement (from 29.6 ± 6.1 to 33.4 ± 6.0). Generalized estimating equations were used to investigate the interactions between the groups and time (pretest vs. posttest). The PWOP scores revealed that the experimental group demonstrated a significant 2.1-point (95% CI = 0.1 to 4.1) improvement in their posttest scores relative to their pretest scores (*p* = 0.046; Table 3). The CW scores indicated that the experimental group demonstrated a nonsignificant 0.5-point (95% CI = 2.1 to 3.0) improvement in their posttest scores relative to their pretest scores (*p* = 0.723; Table 3).

## Discussion

To the best of our knowledge, this is the first study to incorporate immersive simulation course into a physiotherapy clinical internship to improve students’ empathy for, attitudes toward, knowledge of, and willingness to care for older adults and individuals with disabilities. The results indicate that the simulation-based, holistic health-care education implemented in this study was feasible and effective.

### Empathy

The JSE-HPS scores indicate that after the intervention, the experimental group demonstrated a 9.4-point improvement (22.1%), whereas the control group exhibited a 3.0-point improvement (7.1%); the difference between the scores of the two groups was 6.4, with the experimental group achieving a 15% higher average score. This finding is in agreement with those of studies in which simulation teaching interventions are applied for medical, nursing, and pharmaceutical students, with the studies reporting that the interventions, which had durations ranging from 2 to 12 h, improved empathy by 0.7–9.68 points (5%–16.3%) [29–33]. A 22.1% improvement was noted in the present study. A physiotherapist exhibiting empathy is a crucial factor influencing the success of therapy [34]. Moreover, empathy improves the quality of care and patient health outcomes [35, 36]. A potential reason for the improvement in empathy in the present study is the simulation course having been supplemented with an after-class knowledge-sharing component, which may have further enhanced participants’ empathy.

### Attitude

According to the KAOPS scores after the intervention, the experimental group demonstrated a 13.8-point improvement (14.7%), whereas the control group exhibited a 6.2-point improvement (6.1%). Furthermore, according to the ATES scores, the experimental group demonstrated a 5.4-point improvement (8.8%), whereas the control group exhibited a 1.8-point improvement (2.9%). These findings are consistent with those of other studies, which have reported that simulation teaching improved attitudes by 0.32–19.6 points (4%–15.4%) [20, 32, 37–41]. Attitudes can enhance the quality of care, patients’ functional independence, and willingness to care for older adults [42, 43]. The different levels of improvement reported in the aforementioned studies can be attributed to differences in the content of intervention courses, simulation teaching aids, total teaching time (which ranged from 45 min to 2 days), assessment time (ranging from 2 to 4 weeks), and assessment scales that were applied. In the present study, the duration of the simulation-based holistic health-care education intervention was 4 h, and the posttest was conducted in the tenth week, that is, at the midpoint, of the participants’ clinical internships. This design may have enhanced the effectiveness of simulation-based teaching in improving students’ attitudes toward older adults and individuals with disabilities; this improvement may have resulted from the participants reflecting and forming feelings during the simulation.

### Knowledge

The KA scores after the intervention indicate that the experimental group exhibited a 5.0-point improvement (13.4%), whereas the control group demonstrated a 2.3-point improvement (6.4%). The KSOP scores indicate that the experimental group demonstrated a 5.2-point improvement (168%), whereas the control group exhibited a 0.7-point improvement (18.9%). This finding is in agreement with the results of previous studies, which reported that simulation teaching improved knowledge on older adults and individuals with disabilities by 5.9–15.7 points (21.5%–73.8%) [37, 40, 44–46]. Improved knowledge can enhance willingness to care for older adults [47, 48]. In previous studies, the duration of interventions ranged from 50 min to 6 h. Furthermore, various assessment scales were applied, and participants were students (from gerontology health management, medicine, pharmacy, and nursing schools) and household staff. After the simulation-based holistic health-care education intervention was implemented in the present study, the participants exhibited an increase in knowledge on caring for older adults. However, this improvement was limited. A potential reason for this is that of the 4 h of the intervention, only 1 h was allocated to teaching knowledge on care for older adults and individuals with disabilities. The remaining 3 h were allocated to the simulation activity and after-class sharing. Subsequent experimental programs are recommended to increase students’ knowledge or provide information on older adults through online methods.

### Willingness to care

The PWOP scores after the intervention indicate that the experimental group demonstrated a 3.6-point improvement (9.9%), whereas the control group exhibited a 1.3-point improvement (3.7%). The CW scores indicate that the experimental group demonstrated a 3.4-point improvement (10.6%), whereas the control group exhibited a 3.8-point improvement (12.8%). These findings are consistent with those of previous studies, which have reported that simulation teaching improved willingness to provide care by 0.5 points (9.4%–10%) [19, 41]. Studies have also indicated that participating in aged-care-related courses can increase willingness to provide care [49, 50]. A study reported a positive association between older people’s attitudes and motivations for working in aged care [43, 48]. In the present study, after the two groups received standard holistic health-care clinical training for 3.5 h, the experimental group received additional simulation-based holistic health-care education. Both groups exhibited an improved willingness to provide care, which may be attributable to the clinical training that they received for aged care. The differences in scores between the two groups was nonsignificant.

The populations of older adults and individuals with disabilities increase each year. Thus, enhancing patient-centered care, care quality, and empathy for older adults and individuals with disabilities is essential. The present study is the first to incorporate simulation-based holistic health-care education into standard clinical training conducted during a physiotherapy internship. This intervention was implemented to enable interns to understand the effects of physical changes experienced by older adults and individuals with disabilities. To improve the students’ empathy, knowledge, and attitudes, they were asked to experience the constraints encountered by older adults and individuals with disabilities in their daily lives through immersive simulation and after-class sharing sessions.

### Recommendations for follow-up studies

First, the experimental group in the present study participated in both face-to-face teaching and a simulation activity. Future studies can include more intervention groups to investigate the effects of face-to-face teaching intervention when it is implemented alone. Second, the duration of the simulation activity was approximately 2 h. Follow-up studies can increase the amount of time in which students wear simulation suits. For example, students can be asked to wear a restraint belt for an entire day to enable them to understand physical and mental discomfort experienced by individuals with disabilities. Third, four checkpoints were used in the simulation activity. Follow-up studies can add more checkpoints to determine the effects of various interventions. Fourth, we did not include cognitive dysfunction in the situational simulation. Future studies should incorporate dementia into simulation learning. Fifth, the participants enrolled in the present study were physiotherapy interns in their senior year at university. Future studies can enroll students in their freshman, sophomore, or junior year to explore whether the year of study positively affects future internship performance after an intervention.

### Study limitations

The limitations of the present study are as follows. First, a limited number of participants were enrolled. Future studies can increase the sample size in their investigations of whether simulation activities can enhance willingness to provide care. Second, we did not conduct a randomized controlled trial to determine the differences between the experimental and control groups. In addition, the differences associated with participants cohabitating with individuals with disabilities should be considered. Third, future studies can conduct follow-up tests to investigate the effects of interventions over a longer period of time. Fourth, determining minimal clinically important differences (MCIDs) is crucial and clinically beneficial. Future research should determine MCID scores for empathy, attitude, knowledge, and willingness to care.

## Conclusion

The simulation-based, holistic health-care education intervention improved the physiotherapy intern students’ empathy for, knowledge of, and attitudes toward older adults and individuals with disabilities. The results of the present study can serve as a reference for physiotherapy intern units. Simulation teaching can be added to the teaching content for interns to enhance their empathy and improve their care attitudes to thus improve their quality of care and service.

## Data Availability

The data and materials were submitted to Taipei Medical University. The corresponding author can be contacted for additional information.
